# Neurobiological Markers for Predicting Treatment Response in Patients with Bipolar Disorder

**DOI:** 10.3390/biomedicines10123047

**Published:** 2022-11-25

**Authors:** Yen-Ling Chen, Tzu-Hsuan Huang, Pei-Chi Tu, Ya-Mei Bai, Tung-Ping Su, Mu-Hong Chen, Jia-Sheng Hong, Yu-Te Wu

**Affiliations:** 1 Department of Occupational Therapy, I-Shou University, Kaohsiung 840, Taiwan; 2 Institute of Biophotonics, National Yang Ming Chiao Tung University, Taipei 112, Taiwan; 3 Department of Medical Research and Education, Taipei Veterans General Hospital, Taipei 112, Taiwan; 4 Department of Psychiatry, Taipei Veterans General Hospital, Taipei 112, Taiwan; 5 Division of Psychiatry, Faculty of Medicine, National Yang Ming Chiao Tung University, Taipei 112, Taiwan; 6 Institute of Philosophy of Mind and Cognition, National Yang Ming Chiao Tung University, Taipei 112, Taiwan; 7 Department of Psychiatry, Cheng-Hsin General Hospital, Taipei 112, Taiwan; 8 Brain Research Center, National Yang Ming Chiao Tung University, Taipei 112, Taiwan

**Keywords:** bipolar disorder, prediction, biomarker, functional connectivity, brain morphology, cytokine

## Abstract

Predictive neurobiological markers for prognosis are essential but underemphasized for patients with bipolar disorder (BD), a neuroprogressive disorder. Hence, we developed models for predicting symptom and functioning changes. Sixty-one patients with BD were recruited and assessed using the Young Mania Rating Scale (YMRS), Montgomery–Åsberg Depression Rating Scale (MADRS), Positive and Negative Syndrome Scale (PANSS), UKU Side Effect Rating Scale (UKU), Personal and Social Performance Scale (PSP), and Global Assessment of Functioning scale both at baseline and after 1-year follow-up. The models for predicting the changes in symptom and functioning scores were trained using data on the brain morphology, functional connectivity, and cytokines collected at baseline. The correlation between the predicted and actual changes in the YMRS, MADRS, PANSS, and UKU scores was higher than 0.86 (*q* < 0.05). Connections from subcortical and cerebellar regions were considered for predicting the changes in the YMRS, MADRS, and UKU scores. Moreover, connections of the motor network were considered for predicting the changes in the YMRS and MADRS scores. The neurobiological markers for predicting treatment-response symptoms and functioning changes were consistent with the neuropathology of BD and with the differences found between treatment responders and nonresponders.

## 1. Introduction

Bipolar disorder (BD) is one of the leading causes of disability, and the lifetime prevalence of BD type I and type II globally is 0.6% and 0.4%, respectively [1]. The longitudinal course of BD is characterized by recurrent episodes of mania, hypomania, or major depression [2]. Moreover, the interepisode duration after the third episode is shorter than that between the first three episodes. Subsequently, the patient suffers one additional episode per year on average [3]. During a relapse of mood episodes, patients may experience progressive neuropathological changes [4,5], cognitive function deterioration [6,7], and functional impairment [8,9]. Therefore, the prediction of treatment responses as early as possible is essential for making optimal treatment decisions to protect against continuously remaining diminished clinical performance.

Because BD is closely related to brain structure and function [10,11], studies have investigated the replicable predictors of treatment responses in BD by using neuroimaging data. The predictive potential of neuroimaging markers has been investigated by using the baseline status or by analyzing changes from a period of treatment to determine treatment responses and explore the disease mechanism. Changes in the neuroimaging data of patients after treatment have been observed, and the data of treatment nonresponders differ from those of responders [12,13,14]. Moreover, in a previous study, more severe structural and functional imaging changes were found in treatment nonresponders than in treatment responders; these changes were highly correlated with symptoms or functional outcomes [15]. In addition to group-level studies, studies on the prediction of symptoms and functioning in individuals have been conducted. For example, Sartori et al. [16] found a moderate correlation (*r* = 0.59) between the functioning assessment and baseline gray matter volumes, including those of the frontal cortex. Furthermore, Fleck et al. [17] classified patients as responders or nonresponders with 100% accuracy and predicted the treatment response with respect to changes in symptoms with at least 87.9% accuracy. In addition, by using brain-network-based analysis, Wang et al. [18] found significant correlations between the estimated and observed scores of positive symptoms (*r* = 0.35) and mania (*r* = 0.51) in patients with BD who experienced psychosis; these scores were predicted using the between-network connectivity of the visual network, default mode network (DMN), and salience network.

Because inflammatory dysregulation also occurs in patients with BD, cytokines and other inflammatory proteins have attracted increased attention as potential regulators in mood, activity control, and energy [19,20]. Studies have demonstrated that patients with BD have higher cytokine levels than healthy controls do [21,22]. Furthermore, inflammatory pathways are involved in the neuroprogression of BD. These pathways have been suggested as the target of treatment [23]. In a previous study, acutely ill patients were found to have lower cytokine levels than chronically ill patients did [24]. Moreover, the levels of cytokines, including interleukin (IL)-6, tumor necrosis factor (TNF)-α, high-sensitivity C-reactive proteins (CRPs), IL-1 receptor antagonist (IL-1RA), and soluble IL-2 receptor (sIL-2R), in patients decreases after pharmacological treatment [24,25]. The remediation of inflammation and increased anti-inflammatory biomarkers may aid the evaluation of the response to treatments for acute illnesses. Guloksuz et al. [26] demonstrated that compared with good lithium responders, patients with BD who exhibited a poor response had higher levels of TNF-α. Thus, neuroimaging and inflammatory biomarkers have the potential to predict treatment responses to BD.

Although research on neurobiological predictors of treatment response or nonresponse in patients with BD is increasing, no formal definition of treatment resistance exists. Different definitions exist regarding the distinction between responders and nonresponders [14,17]. Hence, an approach for directly predicting symptom and functioning scores is required. In addition, several studies have used cross-sectional data, rather than longitudinal data, to predict symptom scores [16,18]; however, longitudinal data are more likely to indicate cause–effect relationships. Moreover, although Fleck et al. [17] successfully predicted the decrease in symptoms by using longitudinal data, the sample size of their study was small; thus, the generalization error may have been large [27]. Therefore, the present study aimed to use potential predictors from structural or functional neuroimaging and inflammatory biomarkers for predicting the change in symptom and functioning scores during one year. We used the change in scores as the responses to treatment during a year. Taking advantage of the advances in machine learning techniques, we established nonlinear, multidimensional prediction models with a feature selection method to avoid the “curse of dimensionality” for enhancing the development of prognosis and predicting the treatment response to BD.

## 2. Materials and Methods

### 2.1. Participants

In this study, we recruited 61 outpatients with BD (including type I and type II) from Taipei Veterans General Hospital in Taiwan for predicting the changes in symptom and functioning scores. All the patients were stable and were not in the acute stage. The diagnosis of each patient was confirmed by an experienced physician according to the Diagnostic and Statistical Manual of Mental Disorders, Fifth Edition. Patients with neurological illnesses or any other disorders that affect cerebral metabolism, patients with a history of substance abuse or substance dependence over the past 6 months, and patients that suffered from a head injury with a documented sustained loss of consciousness or neurological sequelae were excluded from this study. The clinical assessments of the patients with BD in the analysis for predicting the changes in symptom and functioning scores involved the use of the Young Mania Rating Scale (YMRS), Montgomery–Åsberg Depression Rating Scale (MADRS), Positive and Negative Syndrome Scale (PANSS), UKU Side Effects Rating Scale (UKU), Personal and Social Performance Scale (PSP), and Global Assessment of Functioning scale (GAF). In addition to the baseline symptom and functioning scores, the 1-year follow-up (ranging from 0.5 to 1.2 years) scores were investigated. The investigation was conducted according to the latest version of the Declaration of Helsinki. All the participants gave written informed consent prior to their participation after the experimental procedures were fully explained to them. The present study was approved by the Research Ethics Committee of Taipei Veterans General Hospital.

### 2.2. Acquisition of Resting-State Functional and Structural Magnetic Resonance Imaging Images and Inflammatory Biomarkers

Functional and structural scans were conducted at the Taipei Veterans General Hospital by using a 3.0-T GE magnetic resonance imaging (MRI) scanner (GE Healthcare Life Sciences, Little Chalfont, Milton Keynes, UK) with a quadrature head coil. Resting-state functional images were obtained using a T2 *-weighted gradient-echo, echo-planar sequence (repetition time [TR] = 2500 ms, echo time [TE] = 30 ms, flip angle [FA] = 90°, and voxel size = 3.5 mm × 3.5 mm × 3.5 mm). A total of 200 MRI volumes from each subject were obtained with their eyes closed. A functional whole-brain image volume consisted of 43 interleaved horizontal slices, all of which were parallel to the intercommissural plane. Furthermore, the anatomical whole-brain image volumes were obtained using a sagittal magnetization-prepared rapid acquisition gradient-echo three-dimensional T1-weighted sequence (TR = 2530 ms, TE = 3 ms, echo spacing = 7.25 ms, FA = 7°, field of view = 256 mm × 256 mm, and voxel size = 1 mm × 1 mm × 1 mm) to provide anatomical features for the prediction and to achieve efficient spatial registration and localization of brain activity. This strategy allowed for the better correction of any anatomical differences that might have affected the interpretation during functional analysis.

In addition, cytokines and other inflammatory proteins of the participants, including the soluble IL-6 receptor (sIL-6R), sIL-2R, CRP, P-selectin, monocyte chemoattractant protein-1 (MCP-1), and TNF receptor-1 (TNF-R1), were assayed using enzyme-linked immunosorbent assay kits (R&D systems, Minneapolis, MN, USA).

### 2.3. Preprocessing for Resting-State Functional and Structural MRI Data

The resting-state functional imaging data were preprocessed, and subsequent analyses were performed using Statistical Parametric Mapping (SPM12, Wellcome Institute of Neurology, University College London, London, UK, https://www.fil.ion.ucl.ac.uk/spm/ (accessed on 13 January 2020)) executed in MATLAB 2019b (MathWorks, Natick, MA, USA). The following steps were included in the preprocessing of the images. First, the initial eight volumes were excluded. The slice-dependent time shifts were compensated for timing differences between slices, after which we corrected for head motion and excluded participants with a framewise displacement of >0.2. The functional imaging volumes were then coregistered with their corresponding anatomical images, and spatial normalization was performed into the Montreal Neurological Institute space by using a nonlinear warping algorithm with resampling at a voxel size of 3 mm × 3 mm × 3 mm. Then, spurious data were regressed out by using the Friston 24-parameter model, and the data included white matter signals, cerebrospinal fluid signals, and global signals. Finally, the image data were filtered using a bandpass filter (between 0.01 and 0.08 Hz). Subsequently, smoothing was conducted using a 4-mm full-width at half-maximum Gaussian kernel. Furthermore, participants with considerable head motion (mean framewise displacement of >0.2), which is a source of noise and artifacts, were not considered after the aforementioned procedures. A total of 53 patients with BD were included in the subsequent stages of the experiments. In addition, the cortical and subcortical structures from the structural imaging data were determined using FreeSurfer (version 6.0, https://surfer.nmr.mgh.harvard.edu (accessed on 23 January 2017)) with the following procedures: affine registration with the MNI305 space, B1 bias field correction, skull-stripping, cortical surface reconstruction, gray and white matter segmentation, high-dimensional nonlinear alignment to the MNI305 template, and brain region labeling. Moreover, instead of using the watershed algorithm in FreeSurfer, a more precise skull-stripping method, namely HD-BET [28], which is based on an artificial neural network, was employed.

### 2.4. Feature Extraction

After the preprocessing of the resting-state functional imaging data, functional connectivity was determined according to Shen’s whole-brain functional-connectivity-based atlas to parcellate the whole brain into 268 regions. These regions were then categorized into eight networks: the medial frontal network (MFN), frontoparietal network (FPN), DMN, subcortical and cerebellar regions (SC), motor network (MON), visual I network (VisI), visual II network (VisII), and visual association network (VA). Then, the correlation between each of the aforementioned pairs of regional time series across the 268 regions was estimated using Pearson’s correlation coefficient and converted using Fisher’s r-to-z transformation. Consequently, the functional networks of each patient were formed by 268 × 268 normalized, symmetric correlation matrices. In addition, in the structural imaging data, the volume of the subcortical regions and the volume and thickness of the cortical regions with the Desikan–Killiany atlas were obtained using FreeSurfer. Furthermore, all the neurobiological features, including functional connectivity, volume, thickness, and inflammatory biomarkers, were controlled by age and gender factors.

### 2.5. Feature Selection and Model Training for Predicting the Differences between the Follow-Up and Initial Symptom and Functioning Scores

**Figure 1:**
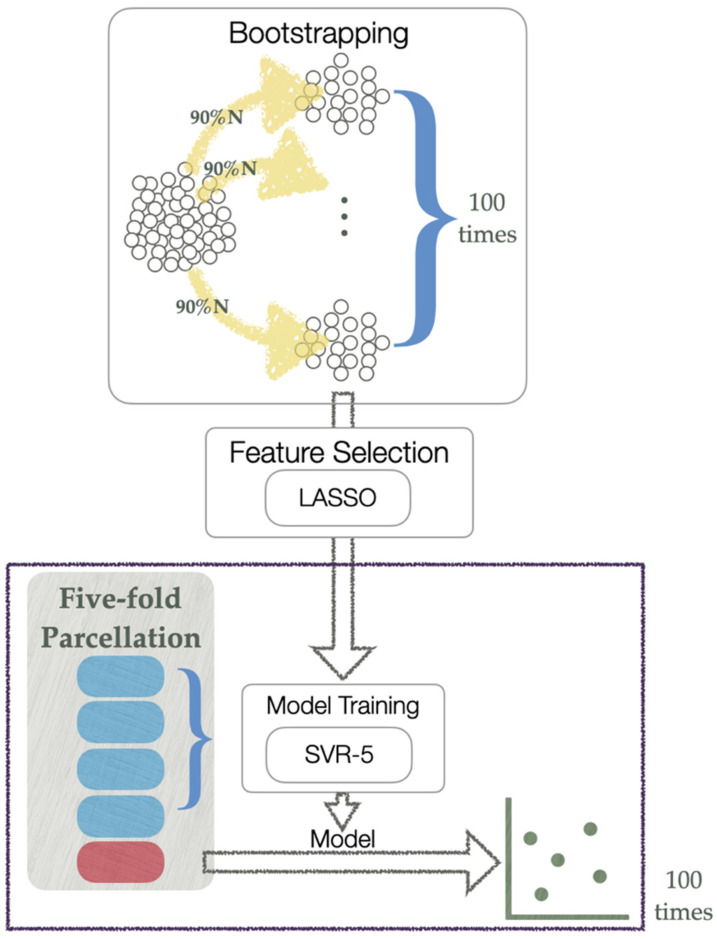
Flowchart of the model prediction procedure. Blue squares are the folds for model training and red square is the fold applied to the trained model. LASSO, least absolute shrinkage and selection operator. SVR-5, support vector machine with five-fold cross-validation.

To establish models for predicting the changes in the symptom and functioning scores, the differences during the 1-year interval of the clinical assessments, including the YMRS, MADRS, PANSS, UKU, PSP, and GAF scores, were calculated and used as responses separately for the prediction models. The functional and structural imaging data and cytokines were used as predictors. Moreover, because the features were in high-dimensional space, we used the least absolute shrinkage and selection operator (LASSO) to investigate the main predictors of the changes in the symptom and functioning scores before model training (see Figure 1) by selecting variables with nonzero coefficients. To generate more generalized features, we bootstrapped the data of 90% of the 53 patients (i.e., 48 patients) 100 times and applied the LASSO to each subsample of patients. The features selected frequently as the variables with nonzero coefficients were used as the predictors of the models. To avoid the “curse of dimensionality,” the number of selected features for each symptom and functioning score was similar to the number of patients (i.e., 53 patients). In addition, to meet the requirements of the LASSO algorithm, the features were standardized prior to feature selection. Next, the models for predicting the change in the score of each clinical assessment for the patients with BD were established 100 times with five-fold nested cross-validation, which helped make the model robust. In the outer loop of nested cross-validation, the patients were randomly separated. Four folds were used as the training set, and one fold was used as the test set. Then, in the inner loop, support vector regression (SVR) was performed by using the training set, which was trained using five-fold cross-validation to estimate the optimal hyperparameters. The predicted values were obtained after all five test parcels were applied to each trained model. Subsequently, the Pearson’s correlation coefficients between the actual and predicted values were examined to determine the performance of prediction models. The mean performance of 100 nested cross-validations was measured.

### 2.6. Effects of Confounding Variables on the Prediction

The effects of illness duration, baseline symptom and functioning scores, and medication were examined by investigating the relationships between these potential confounding variables and the features selected through the LASSO based on bootstrapping. The confounding effects were examined using Pearson’s correlation coefficients for continuous variables and independent sample *t* tests for categorical variables, including patient groups that had and had not taken atypical antipsychotics, patient groups that had and had not taken antidepressants, and patient groups that had and had not taken mood stabilizers. Moreover, because head motion may produce spurious functional connectivity even after head motion correction, the relationships between mean framewise displacement and the selected features were also estimated.

## 3. Results

The demographic and clinical information of the patients that participated in this study is presented in Table 1. As presented in Table 1, only the change in the MADRS score was significant from the baseline.

### 3.1. Prediction Model Performance for the Changes in Symptom and Functioning Scores of the Patients

**Figure 2:**
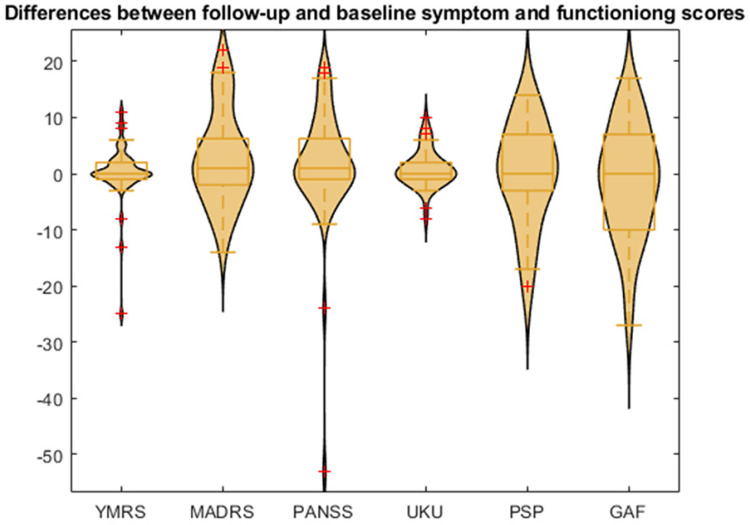
Distribution of the changes in symptom and functioning scores (follow-up score minus baseline status score). The yellow boxes are box-plot of the changes in scores, and the red crosses are outliers. YMRS: Young Mania Rating Scale; MADRS: Montgomery–Åsberg Depression Rating Scale; PANSS: Positive and Negative Syndrome Scale; UKU: UKU Side Effects Rating Scale; PSP: Personal and Social Performance Scale; GAF: Global Assessment of Functioning Scale. The red crosses represent the outliers in the distribution.

The regression models for separately predicting the changes in symptom and functioning scores within a 1-year interval, namely the YMRS, MADRS, PANSS, UKU, PSP, and GAF scores, were developed on the basis of functional connectivity, morphological features, and cytokines. All the predicted changes in the YMRS, MADRS, PANSS, UKU, PSP, and GAF scores were significantly correlated with the actual changes after correcting for false discovery rate (*q* < 0.05). Moreover, as indicated in Table 2, the models of the MADRS and UKU clinical assessments could explain more than 75% of the variance in the response around their means when using the five-fold nested cross-validation of the SVR models and the features selected by the LASSO. However, the models predicting the changes in scores of the YMRS, PANSS, PSP, and GAF did not perform well. Nevertheless, as displayed in Figure 2, strong outliers, namely the values that were three interquartile range (IQR) values lower than the first quartile or three IQR values higher than the third quartile, were observed in the scores of the YMRS (two scores with a considerably negative change) and PANSS (one score with a considerably negative change). To investigate the effects of outliers on regression performance, the regression models for the changes in the YMRS and PANSS scores were retrained after removing the strong outliers. Consequently, the mean correlation coefficients of both the YMRS and PANSS scores increased to more than 0.88, and their regression models could explain more than 75% of the variance in the responses (Table 2).

### 3.2. Features Selected for the Models Developed for Predicting the Changes in Symptom and Functioning Scores

**Figure 3:**
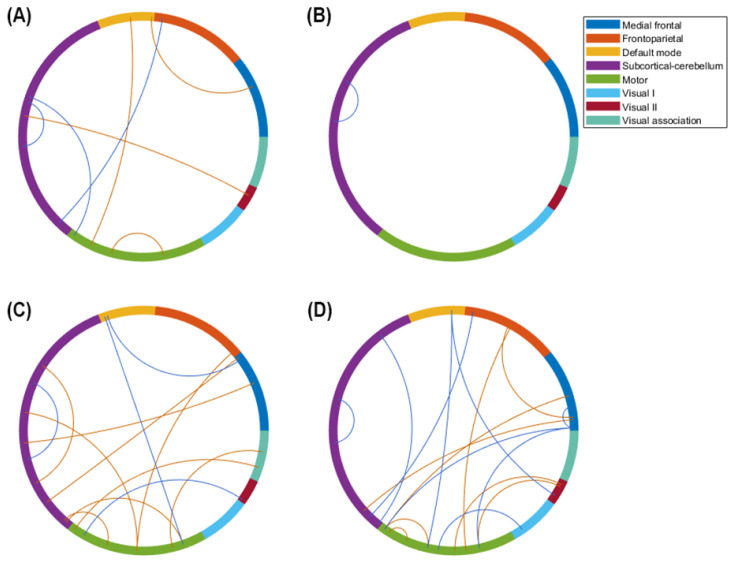
Major features of the prediction models for the changes in symptom and functioning scores. By using the least absolute shrinkage and selection operator 100 times, we bootstrapped the data of 90% of the patients and the selected features. The number of features selected was similar to but fewer than the sample size for training the support vector regression models. The illustration uses the eight networks of Shen’s 268-region parcellation. (**A**) Major features of the regression for the Montgomery–Åsberg Depression Rating Scale. (**B**) Major features of the regression for the UKU Side Effects Rating Scale. (**C**) Major features of the regression for the Young Mania Rating Scale after the removal of strong outliers. (**D**) Major features of the regression for the Positive and Negative Syndrome Scale after the removal of strong outliers. The red line represents the connectivity that is positively correlated with the changes in symptom and functioning scores, and the blue line represents the connectivity that is negatively correlated with the changes in symptom and functioning scores.

During the process of feature selection, 42, 43, 44, 49, 51, and 70 features were frequently selected when using the LASSO for the YMRS, MADRS, PANSS, UKU, PSP, and GAF scores, respectively. Only for the GAF assessment was the number of selected features higher than the number of participants because all the nonzero coefficients were repeated less than 10 times during 100 iterations of the LASSO. All the features used for predicting the changes in symptom and functioning scores were selected on the basis of functional connectivity. No features were selected on the basis of morphological features or cytokines. Moreover, the major features of each regression model were defined as the features that were selected more than 30 times. Figure 3 shows the major features of the prediction models that performed well, namely the models for the changes in the MADRS, UKU, YMRS, and PANSS. The models for the changes in the YMRS and PANSS scores only performed well after the removal of the strong outliers. As displayed in Figure 3, the major features of the MADRS were distributed mostly in the SC, MON, and DMN, and those of the UKU were distributed only within the SC. Furthermore, the functional connections, which were the major features of the YMRS and PANSS, were mostly distributed in the SC/MON and MON/MFN, respectively. Table 3 lists the functional connectivity of the major features, which are represented as the centroids of parcellated regions. This information is provided because Shen’s parcellation is not restricted by the anatomical brain structure. The remaining features involved in the model training were minor features.

### 3.3. Potential Influence of Various Clinical Confounding Factors on the Regression Performance of the Changes in Symptom and Functioning Scores

After correcting for false discovery rate for multiple comparisons, all the features of the successful prediction models exhibited no significant correlation with illness duration and head motion. In the baseline clinical assessments, one of the major features for the prediction model of the PANSS, namely the functional connectivity between the right postcentral cortex and right superior frontal cortex, was strongly related to the baseline PANSS score. Furthermore, one of the major features for the prediction model of the YMRS, namely the functional connectivity between the left parahippocampus and right caudate, was significantly different between patients who had taken antipsychotics and those who had not. No significant association was found in the other features. These results suggested that few clinical confounding factors affected the regression process.

### 3.4. Relationship between Major Features and Inflammatory Markers

**Figure 4:**
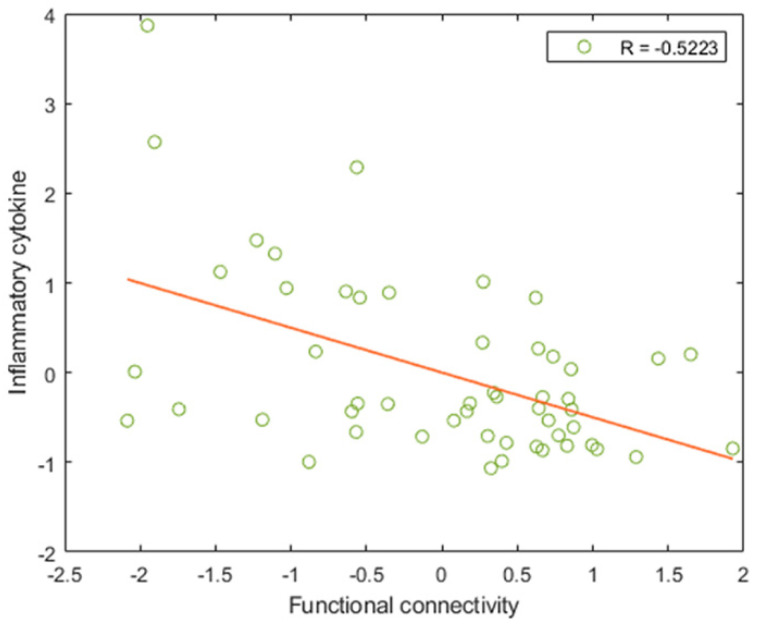
Correlation between inflammatory cytokines and the features of prediction models. The relationship of the C-reactive protein with the functional connectivity between the left postcentral cortex within the motor network and the left superior parietal cortex within the visual association network is also indicated. This connectivity was a minor feature for predicting the changes in the YMRS score. The correlation analyses were conducted by controlling the body mass index and correcting the false discovery rate. The orange line represents the best fit for the data points.

No inflammatory cytokines were identified as major features in all the prediction models. Therefore, because inflammatory cytokines play an essential role in BD, a correlation analysis was conducted on the successful prediction models for further investigating the relationship between the cytokines of the patient group and the features selected during SVR. In terms of the major and minor features used in predicting the changes in MADRS, UKU, YMRS, and PANSS scores, significant correlation was observed between the CRP and a minor feature of the prediction model of the YMRS score, namely the functional connectivity between the left postcentral cortex in the MON and the left superior parietal cortex in the VA (*r* = −0.5223, *q* = 0.0497; see Figure 4) after controlling the body mass index and correcting the false discovery rate. In addition, since the CRP may vary according to mood episodes, the CRP between BD with different mood episodes (i.e., euthymic, hypomania, depressed, and mixed) was compared and showed no significant difference (*p* = 0.4600).

## 4. Discussion

The present study demonstrated that changes in the symptom and functioning scores of patients with BD can be predicted using multiple large-scale networks. The predicted changes in the YMRS, MADRS, PANSS, and UKU scores were highly correlated with the actual changes and explained more than 75% of the variance in response. The results of the present study suggest that changes in the YMRS, MADRS, and UKU scores are mainly predicted by the SC. Changes in the YMRS and MADRS scores are also predicted by the MON. In terms of the centroid of the connections, the regions of basal ganglia contributed to the successful predictions in score changes. Furthermore, the regions of the limbic system, including the thalamus and amygdala, the cerebellum, orbitofrontal cortex, and medial frontal cortex, also contributed to the prediction of the changes in the YMRS, MADRS, and PANSS scores. However, only a few studies have analyzed treatment response prediction in terms of symptom and functioning score changes. All of these studies used morphological patterns, structural integrity, or task-related functional connectivity for the analysis. To the best of our knowledge, this study is the first to use functional connectivity to predict symptom and functioning changes after 1-year of treatment for BD, which should help improve the understanding of neurobiological mechanisms during BD treatment.

The functional connectivity related to the neuropathology of BD is essential in predicting treatment-response symptom and functioning changes. In this study, the major features that can predict score changes in BD were those frequently selected during feature selection in the regions of the basal ganglia, limbic system, the cerebellum, orbitofrontal cortex, and medial frontal cortex. Most of them were regions in the SC and MFN. The functional connectivity of these regions consisted of disruptions found in BD, which is a disorder with emotional dysregulation centered on the prefrontal cortex and amygdala and with abnormal reward processing centered on the prefrontal cortex and striatal regions [29]. Chen et al. [30] identified the abnormalities associated with BD in the parahippocampus, amygdala, hippocampus, putamen, pallidum, caudate, inferior frontal cortex, and lingual gyrus by employing meta-analytical methods. Consequently, the dysconnectivity in BD was considered primarily in the fronto-limbic-striatal regions [31,32], which was also supported by anatomical alterations [29]. In addition to the disruptions found in the cortical and subcortical regions, alterations were noted in the cerebellum, as indicated by the decreased cortical volume [33] and altered functional activity and connectivity [34,35] found in BD. This finding is consistent with those of studies that have indicated that the cerebellum is involved in emotional cue perception and recognition, emotional integration, and emotional modulation [36,37]. Moreover, the disruptions in the aforementioned region were independent of the mood state because abnormalities were present in these regions regardless of the mood state in BD [30,38].

In addition to being related to the neuropathology of BD, the aforementioned regions exhibited differences between treatment responders and nonresponders. Studies have demonstrated that nonresponders show a lower degree of functional connectivity between the amygdala and other fronto-limbic regions [12]; a higher degree of baseline frontoparietal task-related activation [39]; smaller improvements in the fractional anisotropy of the cingulum hippocampus tract, which connects the cingulum and hippocampal regions, after treatment [14]; higher hyperintensity in the white matter of the deep subcortical regions [40]; and a significant increase in the mean diffusivity in the core tracts, which affects the thalamus, caudate, frontal, occipital, temporal, prefrontal, and parietal regions. Furthermore, Wegbreit et al. [12] found that patients whose right amygdala functional connectivity improved after therapy exhibited a decreased YMRS score. Estudillo-Guerra et al. [41] demonstrated that a significant decrease in YMRS scores is associated with perfusion in the orbitofrontal cortex.

In addition to the functional connectivity of the subcortical, cerebellar, and medial frontal regions, the major features were also present in the within- and between-network connectivity of the MON, which was also vital for predicting the symptom and functioning scores of BD. Two studies of the Research Domain Criteria [42] have indicated that the within-network connectivity of the MON and the between-network connectivity of the MON and other subcortical regions, such as the caudate, thalamus, and cerebellum, play an essential role in general psychopathology, cognitive dysfunction, and impulsivity across multiple psychiatric disorders [43]. Moreover, Conio et al. [44] demonstrated that dopaminergic and serotonergic pathways modulate the balance of functional connectivity in sensorimotor regions and the DMN. Moreover, altered biochemical modulation induces dysconnectivity in these networks, which results in the occurrence of different states of BD. In addition to the relationships of BD with the SC and MFN, the relationship between the MON and BD is crucial.

Because the feature for predicting the YMRS, namely the functional connectivity between the left postcentral cortex and left superior parietal cortex, exhibited significant negative correlation with the CRP in the present study, a relationship existed between sensorimotor connectivity and inflammation. This finding was consistent with those of previous studies that have showed that plasma metabolic and inflammatory markers are negatively correlated with functional connectivity involving the reward and motor circuits [45,46]. The aforementioned finding also indicated that the pathways in the reward and motor circuits were sensitive to inflammation. CRP is an acute-phase protein that is produced in response to inflammatory stimuli. It aggravates gliosis, which is a reaction to damage caused to the brain, by releasing interleukins and reducing the synthesis of brain-derived neurotrophic factors, which support neuron survival and growth and thus contribute to neuronal damage [47]. In a previous study, CRP levels were found to be moderately correlated with BD in patients with depression and euthymia but highly correlated with BD in patients with mania [47]. However, no inflammatory markers were selected as features for predicting changes in symptom scores in this study; thus, the obtained results may have been caused by the heterogeneity of the patients. In previous studies, elevated CRP levels have been found only in patients with atypical depression and not in patients with typical depression [48,49].

The present study has several limitations. First, in this study, the changes in symptom and functioning scores were predicted in a naturalistic sample by using neurobiological markers; thus, most of the participants had been treated with medication. However, a significant difference in a major feature was only found when predicting the change in the YMRS score between patients who had and who had not taken antipsychotics. Second, the feature selection process for the prediction of the changes in symptom and functioning scores was conducted for all samples; thus, double dipping was not avoided [50]. No independent test dataset was included during the development of the prediction models. However, the main purpose of the present study was not to develop an effective model for assisting the prognosis of BD but to investigate essential neurobiological markers. Third, only minor changes were observed in the symptom and functioning scores within a 1-year interval among the patients recruited in the present study; therefore, their status was relatively stable. The neurobiological markers used for predicting the score changes in the present study may not be available in patients with pronounced changes in symptom and functioning scores.

## Figures and Tables

**Table 1:** Demographic data (N = 53) for the prediction models of the changes in symptom and functioning scores.

	Baseline (Mean ± SD)	Follow-Up (Mean ± SD)	Change from Baseline (Mean ± SD)	*p*-Value ^‡^
Age	41.06 ± 11.733			
Duration of illness	12.09 ± 9.185			
Sex				
Male (%)	13 (24.5)			
Female (%)	40 (75.5)			
Diagnostic subtype				
Type I	31 (58.5)			
Type II	22 (41.5)			
Medication ^†^				
Atypical antipsychotics (%)	35 (72.9)			
Antidepressants (%)	22 (45.8)			
Mood stabilizers (%)	37 (77.1)			
Clinical assessment				
YMRS	2.91 ± 4.861	3.08 ± 4.636	0.17 ± 5.049	0.8075
MADRS	9.64 ± 10.273	12.47 ± 10.789	2.83 ± 8.485	0.0187
PANSS	38.89 ± 11.265	40.43 ± 10.445	1.55 ± 10.745	0.2993
UKU	3.57 ± 3.208	4.42 ± 3.915	0.85 ± 3.319	0.0682
PSP	71.57 ± 10.231	71.42 ± 9.844	−0.15 ± 8.880	0.9020
GAF	71.75 ± 10.963	70.40 ± 11.394	−1.36 ± 9.909	0.3229

YMRS: Young Mania Rating Scale; MADRS: Montgomery–Åsberg Depression Rating Scale; PANSS: Positive and Negative Syndrome Scale; UKU: UKU Side Effects Rating Scale; PSP: Personal and Social Performance Scale; GAF: Global Assessment of Functioning. ^†^ The medication data of five participants were missing. ^‡^ The *p*-value was obtained using a paired *t* test.

**Table 2:** Regression performance of the models used for predicting the changes in symptom and functioning scores.

Assessments	Mean ± SD of R ^†^	Mean ± SD of R ^†^ after Removing Strong Outliers	R-Squared
YMRS	0.6850 ± 0.1207	0.9187 ± 0.0867	0.8440
MADRS	0.8662 ± 0.0867		0.7503
PANSS	0.6166 ± 0.0714	0.8887 ± 0.0784	0.7898
UKU	0.8861 ± 0.0649		0.7852
PSP	0.8533 ± 0.0971		0.7281
GAF	0.8595 ± 0.0770		0.7387

YMRS: Young Mania Rating Scale; MADRS: Montgomery–Åsberg Depression Rating Scale; PANSS: Positive and Negative Syndrome Scale; UKU: UKU Side Effects Rating Scale; PSP: Personal and Social Performance Scale; GAF: Global Assessment of Functioning. ^†^ Pearson’s correlation coefficient.

**Table 3:** Major features of the prediction models for the changes in symptom and functioning scores.

Regions 1 (with Region Label)					Regions 2 (with Region Label)			
YMRS								
67	Right fusiform gyrus	in	VA	–	24	Right supplementary motor area	in	MON
76	Right lingual gyrus	in	VisII	–	33	Right precentral cortex	in	MON
158	Left postcentral cortex	in	MON	–	4	Right superior orbitofrontal cortex	in	FPN
158	Left postcentral cortex	in	MON	–	135	Left inferior orbitofrontal cortex	in	SC
179	Left postcentral cortex	in	MON	–	177	Left superior parietal cortex	in	VA
224	Left middle cingular cortex	in	SC	–	153	Left inferior orbitofrontal cortex	in	MFN
225	Left precuneus	in	DMN	–	194	Left inferior temporal cortex	in	MFN
227	Left posterior cingular cortex	in	DMN	–	189	Left middle temporal pole	in	MON
233	Left parahippocampus	in	SC	–	123	Right caudate	in	SC
249	Left lobule VIII of cerebellum	in	SC	–	114	Right lobule VI of cerebellum	in	SC
257	Left caudate	in	SC	–	219	Left anterior cingular cortex	in	MFN
266	Left medulla	in	SC	–	51	Left middle temporal pole	in	MON
267	Left pons	in	SC	–	189	Left middle temporal pole	in	MON
MADRS								
90	Right precuneus	in	DMN	–	37	Right insula	in	MON
126	Right thalamus	in	SC	–	24	Right supplementary motor area	in	MON
156	Left inferior triangular frontal cortex	in	MFN	–	3	Right rectus	in	DMN
172	Left postcentral cortex	in	MON	–	60	Right inferior temporal cortex	in	MON
212	Left superior occipital cortex	in	VisII	–	133	Right pons	in	SC
221	Left anterior cingular cortex	in	SC	–	128	Right thalamus	in	SC
264	Left thalamus	in	SC	–	242	Left crus II of cerebellum	in	FPN
PANSS								
24	Right supplementary motor area	in	MON	–	10	Right superior medial frontal cortex	in	MFN
39	Right postcentral cortex	in	MON	–	26	Right superior frontal cortex	in	MON
62	Right Heschl’s gyrus	in	MON	–	24	Right supplementary motor area	in	MON
62	Right Heschl’s gyrus	in	MON	–	49	Right angular gyrus	in	DMN
64	Right middle temporal cortex	in	MFN	–	10	Right superior medial frontal cortex	in	MFN
81	Right inferior occipital cortex	in	VisII	–	49	Right angular gyrus	in	DMN
92	Right amygdala	in	MON	–	75	Right superior occipital cortex	in	VisI
112	Right lobule VIII of cerebellum	in	FPN	–	53	Right middle temporal pole	in	MFN
145	Left superior medial frontal cortex	in	MFN	–	24	Right supplementary motor area	in	MON
168	Left insula	in	MON	–	111	Right crus II of cerebellum	in	FPN
174	Left paracentral lobule	in	MON	–	10	Right superior medial frontal cortex	in	MFN
213	Left lingual gyrus	in	VisII	–	174	Left paracentral lobule	in	MON
214	Left infeior occipital cortex	in	VisII	–	161	Left supplementary motor area	in	MON
224	Left middle cingular cortex	in	SC	–	129	Right medulla	in	SC
261	Left putamen	in	SC	–	52	Right middle temporal pole	in	MFN
264	Left thalamus	in	SC	–	242	Left crus II of cerebellum	in	FPN
268	Left pons	in	SC	–	88	Right middle cingular cortex	in	SC
UKU								
136	Left rectus	in	SC	–	120	Right caudate	in	SC

YMRS: Young Mania Rating Scale; MADRS: Montgomery–Åsberg Depression Rating Scale; PANSS: Positive and Negative Syndrome Scale; UKU: UKU Side Effects Rating Scale; DMN: default mode network; FPN: frontoparietal network; MFN: medial frontal network; MON: motor network; SC: subcortical and cerebellar network; VA: visual association network; VisI: visual I network; VisII: visual II network.

## Data Availability

Not applicable.
